# Small-world topology is most efficient for homeostatic neuronal network repair

**DOI:** 10.1186/1471-2202-12-S1-P357

**Published:** 2011-07-18

**Authors:** Ines Derya Steenbuck, Markus Butz, Marvin Ruiter, Arjen van Ooyen

**Affiliations:** 1 Department of Integrative Neurophysiology, Computational Neuroscience Group, VU University Amsterdam 1081HV Amsterdam, The Netherlands

## 

**Figure 1:**
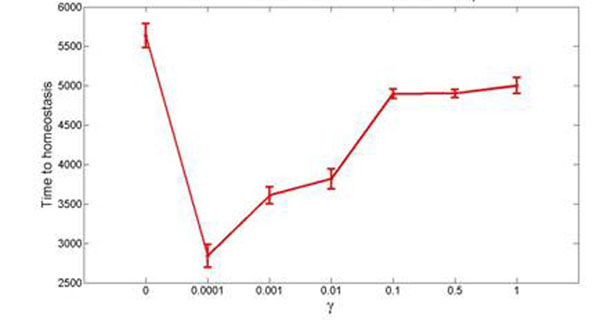
Number of connectivity updates required to completely repair the network as dependent on a small-world parameter γ of the network. Small-world networks go fastest back into homeostasis.

Small-world networks display enhanced signal-propagation speed, computational power, and synchronizability. Neuronal networks in the brain share properties of small-world networks and, in addition, dynamically rewire their connectivity by forming and deleting synapses. It is unclear whether small world networks are best in repairing damages caused by loss of connections and input. Neuronal networks show a reciprocal interaction between topology and the flow of neuronal (electrical) activity they generate. Topology determines the activity flow through the network, whereas on a longer timescale, the flow of activity guides new connections to be formed or existing ones to be removed. Importantly, neurons thereby try to maintain their electrical activity at a certain setpoint (homeostasis of electrical activity). That is, if neurons loose synaptic input due to a lesion, they respond with a local change in connectivity to obtain more connections and activity from other sources. Here we investigate by a computational modelling study based on a model for activity-dependent structural plasticity [1,2], first, how local changes in synaptic connectivity alter global network topology after a circumscribed loss of input; and second, which topologies best support network repair re-establishing homeostasis in electrictal activity of all neurons. We found that reorganizing networks become more random as they form more long-range connections after a loss of input and those neurons losing their input increase their betweenness centrality. Interestingly, an increased randomness and betweenness centrality has recently been found in functional connectivity of ipsilateral cortical and contralateral cerebellar networks following subcortical stroke [3]. As a second important result we found that small-world networks recover fastest (Fig.1), compared to regular and random networks, from a loss of input in the sense that all neurons return to homeostasis in electrical activity. The small-worldness of brain networks may therefore have an evolutionary advantage since those networks are more robust against lesions than regular (and random) networks.
